# Oral versus intravenous administration of cyclophosphamide: a case report

**DOI:** 10.1186/1757-1626-1-395

**Published:** 2008-12-15

**Authors:** Kenneth S Wu, Chung Kwok, Aishin Lok

**Affiliations:** 1 Department of Nephrology, St James's University Hospital, Beckett Street, Leeds, LS9 7TF, UK

## Abstract

**Background:**

Cyclophosphamide, an alkylating agent, has been used as an immunosuppressant in the treatment of various autoimmune disorders and malignancies. It is highly capable of reducing T and B lymphocytes.

**Case presentation:**

Regular blood tests are important to detect bone marrow suppression. The risk of opportunistic infection such as pneumonia secondary to pneumocystic carinii increases drastically with lymphopaenia. Hence, prophylactic co-trimoxazole is frequently prescribed as long as patients are on cyclophosphamide.

**Conclusion:**

The benefits and toxicity risks of intravenous versus oral administration of cyclophosphamide are described in this article.

## Case presentation

A 60-year-old lady presented with 3 months history of non-specific flu-like symptoms, fever, lethargy and polyarthralgia. Urinalysis revealed haematoproteinuria. She was admitted with acute renal failure and found to have a C-ANCA (anti-neutrophil cytoplasmic antibodies) associated vasculitis with high PR3 (proteinase 3). Serum creatinine was >900 umol/L. Renal biopsy revealed multiple cellular creascents. Treatment with Intravenous(IV) monthly pulsed cyclophosphamide(CYC), steroid and plasma exchange(PE) were carried out. Although she had a prolonged hospital admission, she eventually became dialysis independent. Her serum creatinine was 195 umol/L at 3 months after her initial treatment.

## Discussion

Wegener's granulomatosis (WEG) is a type of serious vasculitis. Without treatment, mortality is as high as 90% at 2 years. Presenting features include corneal ulceration, episcleritis, abnormalities in the respiratory tract, proptosis, diplopia, mononeuritis multiplex, pulmonary haemorrhage and renal failure. Rhinorrhoea, bloody nasal discharge, oral ulcers, myalgia and polyarthralgia appeared to be the most common presenting complaints.

Cyclophosphamide with glucocorticoid has been frequently used in managing various types of vasculitis including WEG. It is an alkylating agent which impairs DNA replication leading to cell death. It also has the ability to inhibit T cell proliferation. There are serious adverse effects associated with this agent including premature ovarian failure, bone marrow suppression, bladder cancer and hyponatraemia due to SIADH (syndrome of inappropriate antidiuretic hormone secretion).

There has been much interest in the route of administration of CYC in vasculitic disorders. The higher the cumulative dose, the higher the risk of toxicity is. Some studies have reported the risk of infection and lymphopaenia are lower with IV pulsed CYC as the total accumulative dose is much lower [1]. Bladder toxicity also appears to be significantly lower in patients who receive CYC through the IV route. The incident of bladder cancer is around 15% at 15 years [2]. The benefit of MESNA (sodium 2-mercaptoethanesulfonate) to reduce haemorrhagic cystitis and bladder cancer is not supported by all studies. Permanent and premature ovarian failure is a well recognised complication of CYC. The risk of gonadal failure increases with age. One study has shown the risk of ovarian dysfunction is 10% and 60% in women who are younger than 26 years old and those older than 30 years old respectively [3]. Males who receive this agent are also at risk of infertility. Although more challenging in women, gonadal cryopreservation can be performed in both genders. Gonadotropin-releasing hormone agonist has been shown to be useful to reduce the risk of premature ovarian failure.

## Conclusion

Cyclophosphamide can be a very useful drug given in the right setting with sufficient monitoring. There is increasing evidence to support IV CYC therapy is equally as effective as oral therapy and is associated with less toxicity in patients with systemic vasculitis.

## Abbreviations

CYC: Cyclophosphamide; IV: Intravenous; WEG: Wegener's granulomatosis vasculitis.

## Consent

Written consent was obtained from patient. This is available for review by the editor of the journal.

## Competing interests

The authors declare that they have no competing interests.

## Authors' contributions

KW obtained consent and treated the patient. CK and AL involved in preparing the manuscript and researching information.

## References

[B1] de Groot K, Adu D, Savage CO, EUVAS (European vasculitis study group) (2001). The value of pulse cyclophosphamide in ANCA-associated vasculitis: meta-analysis and critical review. Nephrology Dialysis Transplantation.

[B2] Talar-Williams C, Hijazi YM, Walther MM, Linehan WM, Hallahan CW, Lubensky I, Kerr GS, Hoffman GS, Fauci AS, Sneller MC Cyclophosphamide-induced cystitis and bladder cancer in patients with Wegener granulomatosis. Ann Intern Med.

[B3] Boumpas DT, Austin HA, Vaughan EM, Yarboro CH, Klippel JH, Balow JE (1993). Risk for sustained amenorrhea in patients with systemic lupus erythematosus receiving intermittent pulse cyclophosphamide therapy. Ann Intern Med.

